# Pioglitazone attenuates advanced glycation end products‐induced apoptosis and calcification by modulating autophagy in tendon‐derived stem cells

**DOI:** 10.1111/jcmm.14901

**Published:** 2020-01-19

**Authors:** Langhai Xu, Kai Xu, Zhipeng Wu, Zhonggai Chen, Yuzhe He, Chiyuan Ma, Safwat A. A. Moqbel, Jisheng Ran, Caihua Zhang, Lidong Wu, Yan Xiong

**Affiliations:** ^1^ Department of Orthopedics Surgery The 2nd Affiliated Hospital Zhejiang University School of Medicine Hangzhou China

**Keywords:** advanced glycation end products, apoptosis, autophagy, pioglitazone, tendon‐derived stem cells

## Abstract

Diabetes mellitus (DM) is one of the prominent risk factors for pathological development and progression of tendinopathy. One feature of DM‐related changes in tendinopathy is accumulation of advanced glycation end products (AGEs) in affected tendons. Pioglitazone (Pio), a peroxisome proliferator‐activated receptor γ agonist, performs a protective effect against AGEs. The present study aimed to investigate the pathogenetic role of AGEs on tendon‐derived stem cells (TDSCs) and to determine the effect of Pio on AGEs‐induced TDSC dysfunctions. Results indicated that AGEs induced TDSC apoptosis as well as compensatory activation of autophagy. Pharmacologic activation/inhibition of autophagy leaded to alleviate/exacerbate apoptosis induced by AGEs. We further confirmed the effect of Pio on autophagy, which ameliorated apoptosis and abnormal calcification caused by AGEs both in vitro and in vivo. Thus, we suggest that Pio ameliorates the dysfunctions of TDSCs against AGEs by promoting autophagy, and we also reveal that Pio is a potential pharmacological choice for tendinopathy.

## 1 INTRODUCTION

Tendinopathy is a frequent, disabling musculoskeletal condition as a result of a chronic imbalance between degeneration and repair in the tendon. Diabetes mellitus (DM), as a systemic metabolic disorder, is an important risk factor for the development and poor prognosis of tendinopathy.^1^, ^2^ Tendon‐derived stem cells (TDSCs) are isolated from tendon tissues and possess the capacity of self‐renewing and differentiating into tendon‐like tissues.^3^ TDSCs can promote tendon repair and regeneration, and maintain tendon homeostasis.^4^ Impaired function of TDSC may account for the structural alternations in DM tendons, which may exacerbate tendon matrix degradation and tendinopathy progression.^5^

Advanced glycation end products (AGEs), kinds of oxidative derivatives resulting from diabetic hyperglycaemia, are known to contribute to the complications of DM by raising intracellular oxidative stress.^6^ Extracellular AGEs induce cellular oxidative stress, inflammation and apoptosis in DM complications such as cardiovascular disease and chronic kidney disease.^7^, ^8^ Meanwhile, studies showed that AGEs can accumulate in long‐lived tissues like tendons and bridge between the free amino groups of neighbouring proteins to form intermolecular crosslinks, which in turn increases tissue stiffness and brittleness.^9^

Pioglitazone (Pio), a peroxisome proliferator‐activated receptor (PPAR) γ agonist, is widely used in clinical practice to treat type 2 diabetes. Recent studies have showed that Pio can also perform anti‐inflammation and anti‐apoptosis effects against AGEs in cardiovascular disease, kidney disease and others.^10^, ^11^, ^12^ There is growing evidence that Pio can enhance autophagy to ameliorate cell damage and tissue injury.^13^, ^14^ Nevertheless, whether pioglitazone can be used to improve tendinopathy in DM is still unknown.

In the present study, we found that AGEs induced apoptosis of TDSC, while cellular autophagy could ameliorate it; Pio improved cellular autophagy and attenuated AGEs‐induced apoptosis and abnormal calcification.

## 2 MATERIALS AND METHODS

### 2.1 Preparation of AGEs‐modified BSA

About 30 mg/mL BSA was incubated with 0.1 M glyceraldehyde in 0.2 M NaPO4 buffer (PH7.4) at 37℃ for 7 days and then dialysed against phosphate‐buffered saline for 2 days to remove the unbonded sugars. Control BSA was incubated in the same conditions without sugars.^15^ Estimation of glycation was assayed by measuring the fluorescence of AGE and non‐glycated BSA solutions with excitation wavelength of 370nm and emission wavelength of 440 nm,^16^ and AGE solution showed 48‐fold stronger fluorescence intensity than that of control BSA solution.

### 2.2 Cell culture

This study was approved by the Institutional Animal Care and Use Committee of Zhejiang University (Hangzhou, China). Achilles tendons were obtained from 3‐week‐old Sprague Dawley (SD) rats (Zhejiang Academy of Medical Sciences Hangzhou, China). After cutting into 1 mm^3^ particles, the tendons were incubated with 0.1% type I collagenase on a horizontal shaker at 37℃ for 3 h to isolate tenocytes. Single‐cell tendon‐derived cells were cultured in 96‐well plates for 7 days, and colonies were collected as passage 0 (P0) and passaged three times prior to use in all experiments. DMEM supplemented with 10% FBS and 100 units/mL penicillin, and 100 μg/mL streptomycin was used to expand single‐cell colonies. Cells were cultured at 37℃ with 5% CO_2_.

### 2.3 Identification of surface markers

Cells were incubated with fluorescent primary antibody on ice in PBS for 60 min, washed 3 times and detected using flow cytometry. The negative control had no fluorescent antibody. The following fluorescent primary antibodies were used: FITC antirat CD29, FITC anti‐rat CD44, PE anti‐rat CD45, PE anti‐rat CD90 (Bioleague).

### 2.4 Multipotency analysis

Cells were incubated in specific differentiation media: adipogenesis with adipogenic induction and maintenance medium (Cyagen Biosciences,) for 14 days, Oil Red staining was used to confirm the differentiation to adipocytes; osteogenesis with osteogenic induction medium (Cyagen Biosciences) for 14 days, Alizarin Red staining was used to confirm the differentiation to osteoblasts; chondrogenesis in pellet culture with chondrogenic induction medium (Cyagen Biosciences) for 21 days, Safranin O staining was used to confirm the differentiation to chondrocytes.

### 2.5 Cell viability analysis

To analyse cytotoxicity of AGEs, Pio on TDSC, CCK‐8 assay was conducted according to the manufacturer's instruction. A total of 5 × 10^3^ cells were seeded in 96‐well plates and treated with different concentrations of AGEs, Pio or both for 24 h. Cells were incubated with 10μl CCK‐8 reagent per well for 3 h and then read at a wavelength of 450 nm with a microplate spectrophotometer.

### 2.6 Apoptosis analysis

A total of 1 × 10^6^ TDSCs (passage 3) were double‐stained using the fluorescent dye annexin V‐FITC/Propidium Iodide (PI) Apoptosis Detection Kit (Keygen Biotech) according to the manufacturer's instructions. Apoptosis cells (annexin V+/PI‐) were detected using flow cytometry.

### 2.7 Western blot analysis

After treatment, TDSCs were washed three times with phosphate‐buffered saline (PBS) and lysed with RIPA for 60 min. Then, the samples were separated via 10% or 15% sodium dodecyl sulphate (SDS)‐polyacrylamide gels and transferred into nitrocellulose membranes. The membranes were blocked with 5% BSA for 1h and cut into sections based on different protein molecular weights. Membranes were incubated with primary antibodies at 4°C overnight. Then, the membranes were incubated with secondary antibodies for 1h and luminesced using Pierce™ ECL Western blotting substrate. The relative amount of proteins was analysed with Quantity One software (Bio‐Rad) and normalized to β‐actin. All assays were performed in triplicate.

### 2.8 Immunofluorescence

TDSCs cultured on 24‐well plates were pretreated with Pio (0, 50 μM) for 1 h and then incubated with different concentration of AGEs for 6 h. After fixation with methanol for 30 min, the cells were permeabilized by PBS containing 0.5% v/v Triton X‐100 for 15 min and blocked with 5% BSA for 1 h. The cells were incubated with primary antibody against LC3B at 4°C overnight, followed by being incubated with fluorescein isothiocyanate‐conjugated secondary antibodies for 1 h. Cell nucleuses were stained with DAPI for 5 min, and then, cells were analysed with a Leica fluorescence microscope.

### 2.9 Senescence analysis

β‐galactosidase Activity Assay (Beyotime Biotechnology, Shanghai, China) was performed to measure cellular senescence according to the manufacturer's instruction. Cells were cultured in β‐galactosidase staining buffer for 24 h and visualized with microscope.

### 2.10 ALP staining

Cells were cultured in 12‐well plates with osteogenic induction medium for 5 days. Cells were fixed with 4% paraformaldehyde for 30 min and subsequently stained by Alkaline Phosphatase Color Development Kit (Beyotime).

### 2.11 Alizarin Red staining

Mineral deposition was assessed by Alizarin Red staining (ARS) (Cyagen Biosciences) after the induction of osteogenic differentiation for 2 weeks. Cells were fixed with 4% paraformaldehyde for 30 min and then incubated with 0.1% solution of Alizarin Red for 10 min at room temperature.

### 2.12 Animal model

Twenty‐four male Sprague Dawley rats (200‐250 g; 6 weeks old) were used and randomly divided into four groups (six rats in each group): TP, TP + AGE, TP + Pio and TP + AGE+Pio. All rats underwent Achilles tenotomy.^17^ Rats in TP + AGE and TP + AGE+Pio groups were injected with 0.1 mL of 40 μg AGE once a week in the region surrounding Achilles tendon, and other two groups were injected with 0.1 mL of 40 μg BSA as control; rats in TP + Pio and TP + AGE+Pio groups were injected with 0.1 mL of 5 nmol Pio twice a week in the region surrounding Achilles tendon, and other two groups were injected with 0.1 mL PBS as control. Six weeks after Achilles tenotomy, rats were euthanized and their limbs were harvested for further study. The study was conducted in accordance with NIH guidelines (NIH Pub No 85‐23, revised 1996), and the protocol was approved by the Ethics Committee of the Second Affiliated Hospital, School of Medicine, Zhejiang University, Hangzhou, China.

### 2.13 Histological analysis

The knee samples were cut into 5 μm sections. These sections were stained with HE staining, modified Masson staining and TUNEL staining, according to the manufacturer's instructions.

### 2.14 Statistical analysis

All data are presented as means ± SDs. One‐way ANOVA with a subsequent post hoc Tukey's test was used for multiple comparisons. The value of *P* < .05 was considered to indicate significant differences.

## 3 RESULTS

### 3.1 Identification of TDSCs

**Figure 1:**
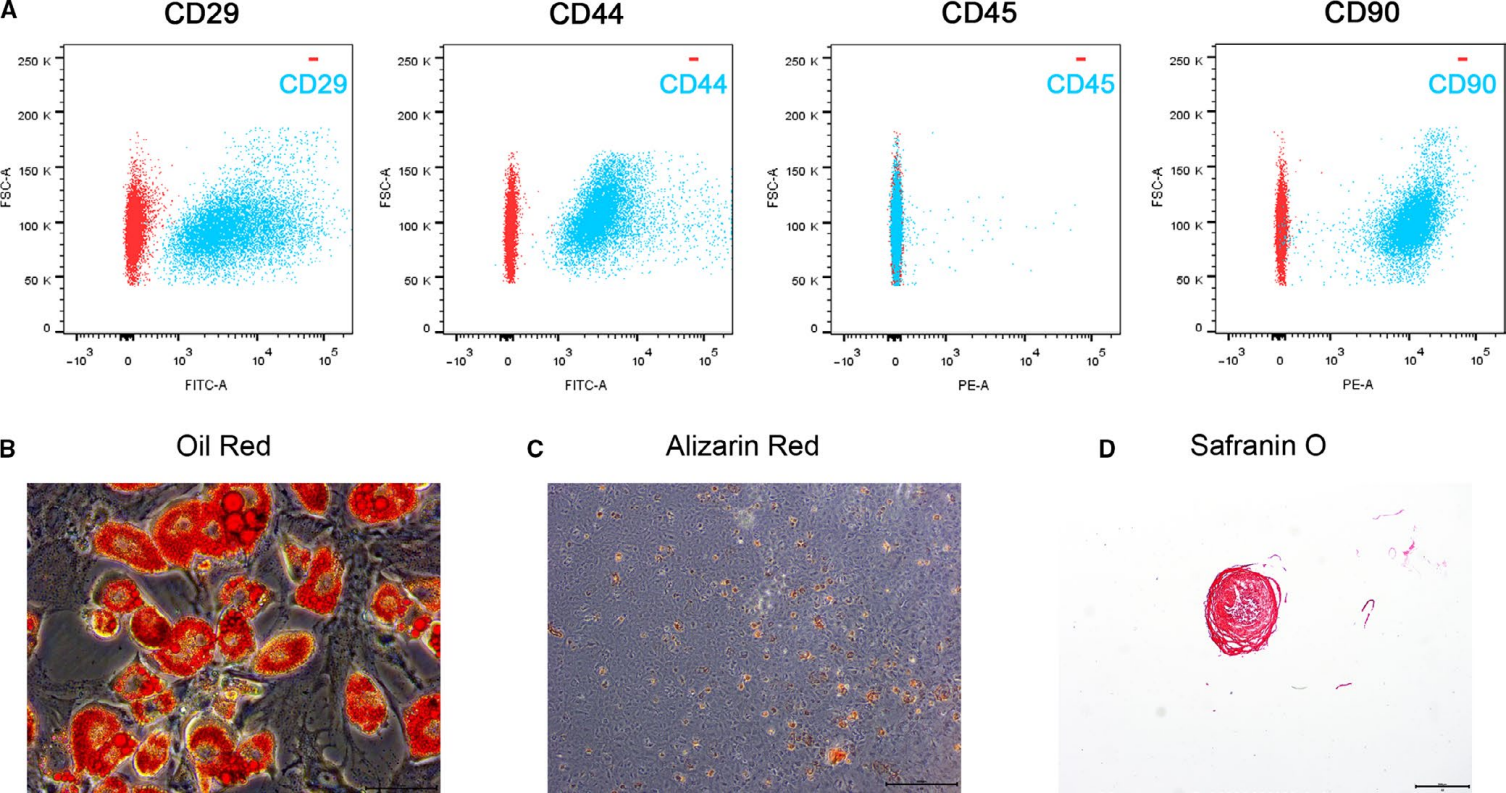
Identification of TDSCs. The surface marker analysis and multipotency analysis were performed to identify the stem status of TDSCs. (A) Representative flow cytometric profiles of TDSCs stained for CD29, CD44, CD45, CD90 (red: control; blue: fluorescent antibody). (B‐D) Differentiation of TDSCs. B (Bar = 50 μm) showed adipogenesis (Oil Red); C (Bar = 500μm) shows osteogenesis (Alizarin Red); D (Bar = 500 μm) showed chondrogenesis (Safranin O)

To identify the stem status of the clonogenic cells, a cell surface marker analysis was performed. Results showed that the clonogenic cells expressed high levels of CD29, CD44, CD90 (stem cell markers), and undetectable level of CD45 (leucocyte marker) (Figure 1A).^18^ Multipotency of the clonogenic cells was also analysed to further elucidate the stem cell properties. Oil Red staining of adipogenic cultures showed significant amounts of newly differentiated adipocytes (Figure 1B). Alizarin Red staining of osteogenic cultures showed calcium deposits within the cell layer (Figure 1C). Safranin O staining of chondrogenic pellet cultures showed positive potential towards chondro‐lineage phenotype (Figure 1D).

### 3.2 AGEs induce apoptosis of TDSCs

**Figure 2:**
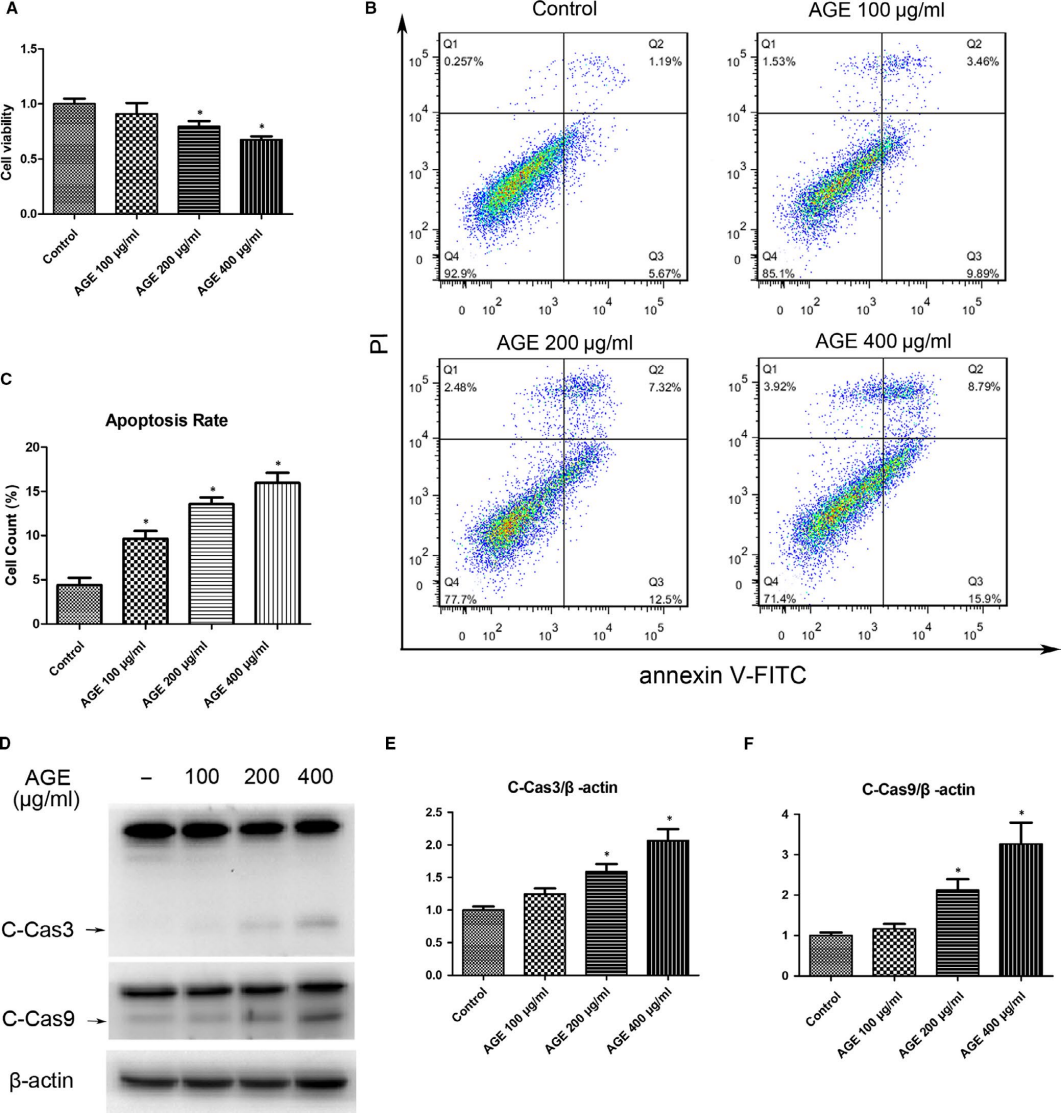
AGEs induce apoptosis of TDSCs. TDSCs were treated with 100, 200 or 400 μg/mL AGEs for 24 h. Cells incubated with 400 μg/mL BSA were used as controls. (A) Cell Counting Kit‐8 results of treated TDSCs. (B, C) Apoptosis was detected using the annexin V‐FITC/PI kit. Data were measured by the FlowJo software. (D‐F) Western blot analysis of the protein expression levels of cleaved caspase‐3 and cleaved caspase‐9 in treated TDSCs. *P < .05

The cell viability of TDSCs decreased with AGEs treatment in a dose‐dependent manner, and significant decreases in cell viability were observed at a concentration of 200μg/ml and 400μg/ml compared to the control group (Figure 2A). The flow cytometry showed that AGEs induced a great degree of apoptosis, and a 15% apoptosis rate was observed at a concentration of 400μg/ml (Figure 2B,C). Western blotting results showed that AGEs significantly increased the expression of cleaved caspase‐3 (C‐Cas3) and cleaved caspase‐9 (C‐Cas9) (Figure 2D‐F). These results suggest that AGEs induce apoptosis of TDSCs.

### 3.3 Autophagy protects cells against apoptosis induced by AGEs

**Figure 3:**
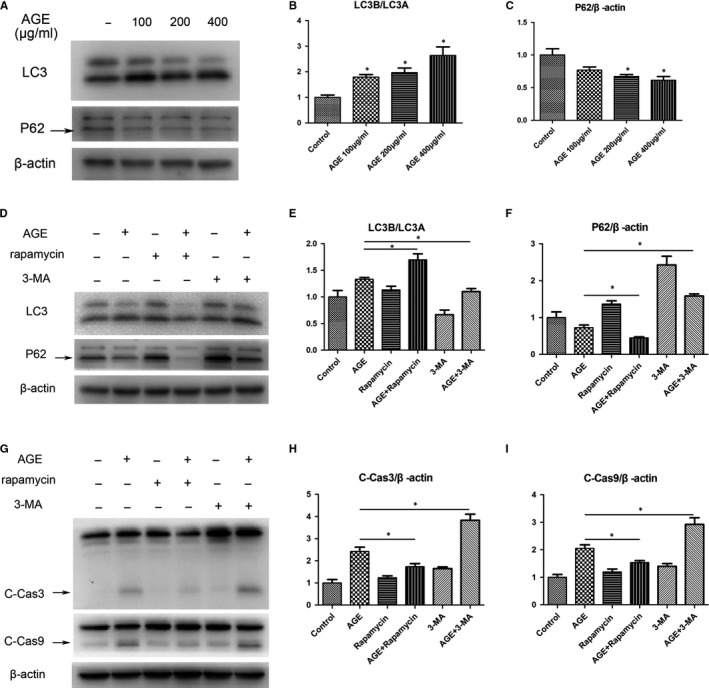
Autophagy protects cells against apoptosis induced by AGEs. TDSCs were treated with 100, 200 or 400 μg/mL AGEs for 6h. Cells incubated with 400 μg/mL BSA were used as controls. (A‐C) Western blot analysis of the protein expression levels of LC3A/B and P62 in treated TDSCs. TDSCs were pretreated with rapamycin or 3‐MA for 2 h and then incubated with AGEs or BSA. (D‐F) Western blot analysis of the protein expression levels of LC3A/B and P62 in TDSCs incubated with AGEs for 6 h. (G‐I) Western blot analysis of the protein expression levels of cleaved caspase‐3 and cleaved caspase‐9 in TDSCs incubated with AGEs for 24 h. *P < .05

Microtubule‐associated protein light chain 3 (LC3) is a biomarker to monitor autophagy, and the ratio of LC3B/LC3A correlates with the number of autophagosomes.^19^ P62/SQSTM1 acts as a link between the ubiquitination and autophagy machineries, and the accumulation of P62 suggests repressed autophagic degradation.^20^ In this study, Western blotting was performed and showed an increase in the ratio of LC3B/LC3A and a decrease in P62 following AGEs treatment, suggesting an increase in cellular autophagy (Figure 3A‐C). To ascertain the impact of autophagy on cellular apoptosis, TDSCs were pretreated with rapamycin (autophagy agonist) or 3‐MA (autophagy antagonist) for 2 h to activate or inhibit cellular autophagy, and then incubated with AGEs. Results showed that the ratio of LC3B/LC3A was up‐regulated with rapamycin pretreated and down‐regulated with 3‐MA pretreated, while P62 was down‐regulated with rapamycin pretreated and up‐regulated with 3‐MA pretreated, suggesting that autophagy level was elevated by rapamycin and repressed by 3‐MA (Figure 3D‐F). C‐Cas3 and C‐Cas9 were showed a decrease with rapamycin pretreated and an increase with 3‐MA pretreated, suggesting that autophagy promotes cell survival against apoptosis (Figure 3G‐I).

### 3.4 Pioglitazone induces autophagy in TDSCs

**Figure 4:**
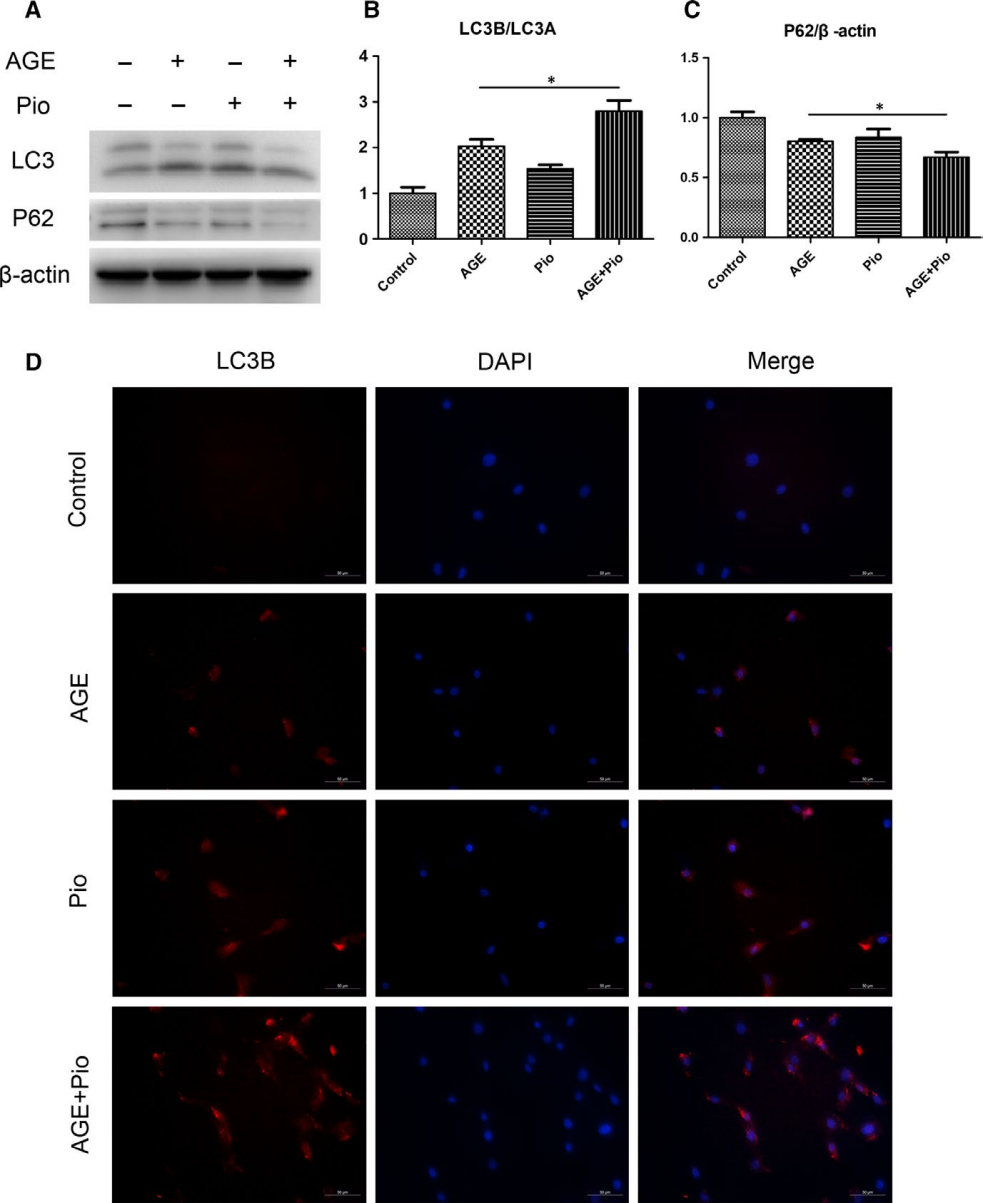
Pioglitazone induces autophagy in TDSCs. TDSCs were pretreated with pioglitazone for 2 h and then incubated with AGEs or BSA for 6 h. (A‐C) Western blot analysis of the protein expression levels of LC3A/B and P62 in treated TDSCs. (D) Immunofluorescence of LC3B in treated TDSCs (red: LC3B; blue: DAPI), Bar = 50μm. *P < .05

Recent study showed that Pio can reduce tissue injury via modulation of autophagy.^13^ To determine whether Pio performed the similar effect on TDSCs, alterations in autophagy were assessed. The CCK8 assay showed that Pio exhibited no significant cytotoxicity to the cultured TDSCs at concentrations of ≤ 100 μM (Figure S1). Western blotting showed that the ratio of LC3B/LC3A was up‐regulated and P62 was down‐regulated with Pio pretreated (Figure 4A‐C). Immunofluorescence results also showed that LC3B fluorescence intensity increased in TDSCs with Pio pretreated (Figure 4D), suggesting that Pio promoted autophagy in TDSCs. We further explored whether the AMPK/mTOR pathway is involved in Pio‐induced autophagy flux. Western blotting was performed and showed that Pio treatment significantly increased p‐AMPK and decreased p‐mTOR, suggesting that Pio activated the AMPK/mTOR pathway to promote autophagy (Figure S2A‐C).

### 3.5 Pioglitazone decreases AGEs‐induced apoptosis in TDSCs

**Figure 5:**
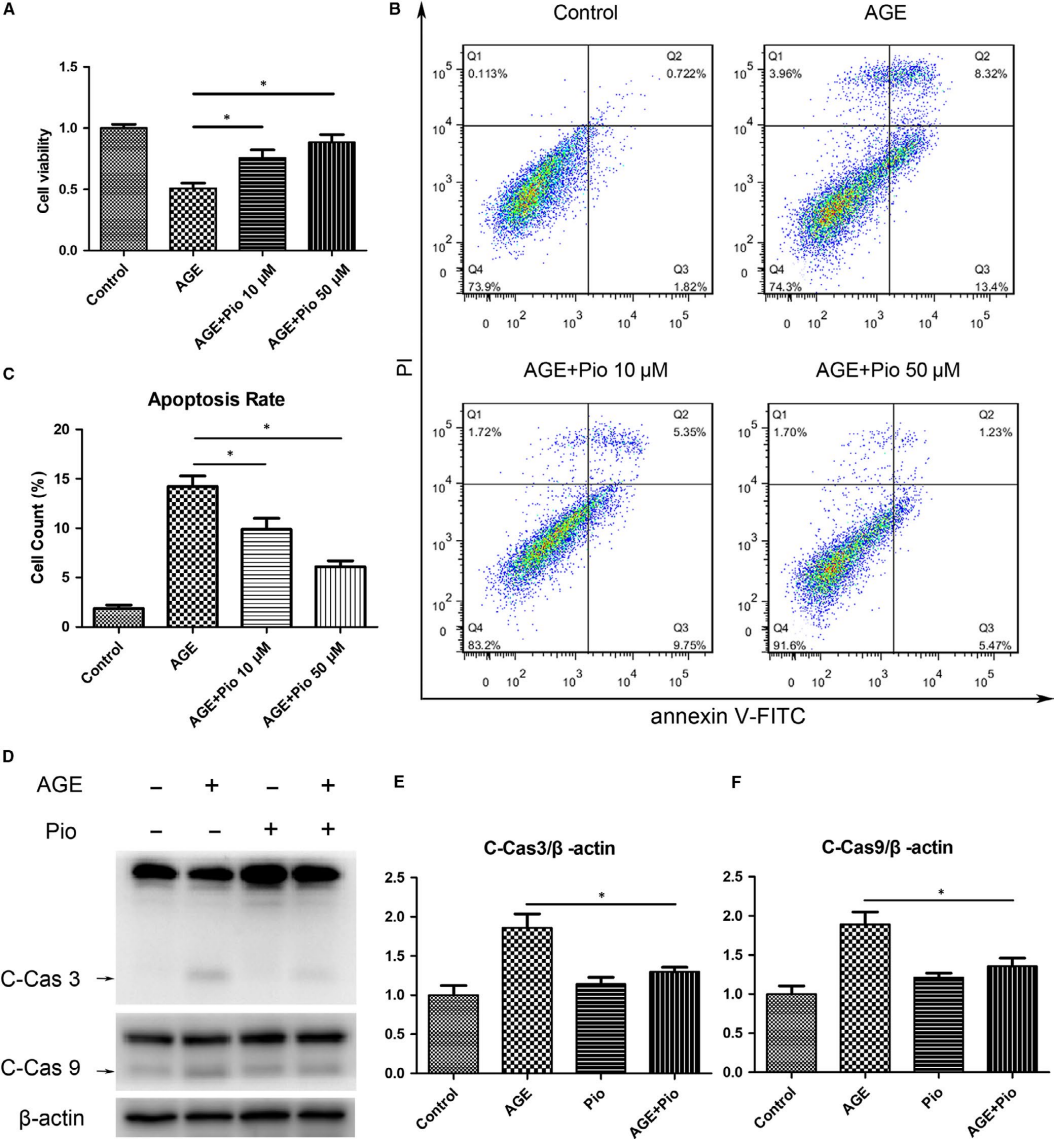
Pioglitazone decreases AGEs‐induced apoptosis in TDSCs. TDSCs were pretreated with pioglitazone for 2 h and then incubated with AGEs or BSA for 24 h. (A) Cell Counting Kit‐8 results of treated TDSCs. (B, C) Apoptosis was detected using the annexin V‐FITC/PI kit. Data was measured by the FlowJo software. (D‐F) Western blot analysis of the protein expression levels of cleaved caspase‐3 and cleaved caspase‐9 in treated TDSCs. *P < .05

To verify whether Pio can protect TDSCs, the CCK8 assay was performed and showed that Pio had a protective effect against AGEs (Figure 5A). The flow cytometry showed that pretreatment with Pio significantly decreased AGEs‐induced apoptosis in TDSCs (Figure 5B,C). Western blotting also showed that pretreatment with Pio significantly attenuated elevated expression of C‐Cas3 and C‐Cas9 induced by AGEs (Figure 5D‐F). These results suggest that Pio decreases AGEs‐induced apoptosis in TDSCs.

### 3.6 Pioglitazone alleviates AGEs‐induced senescence in TDSCs

**Figure 6:**
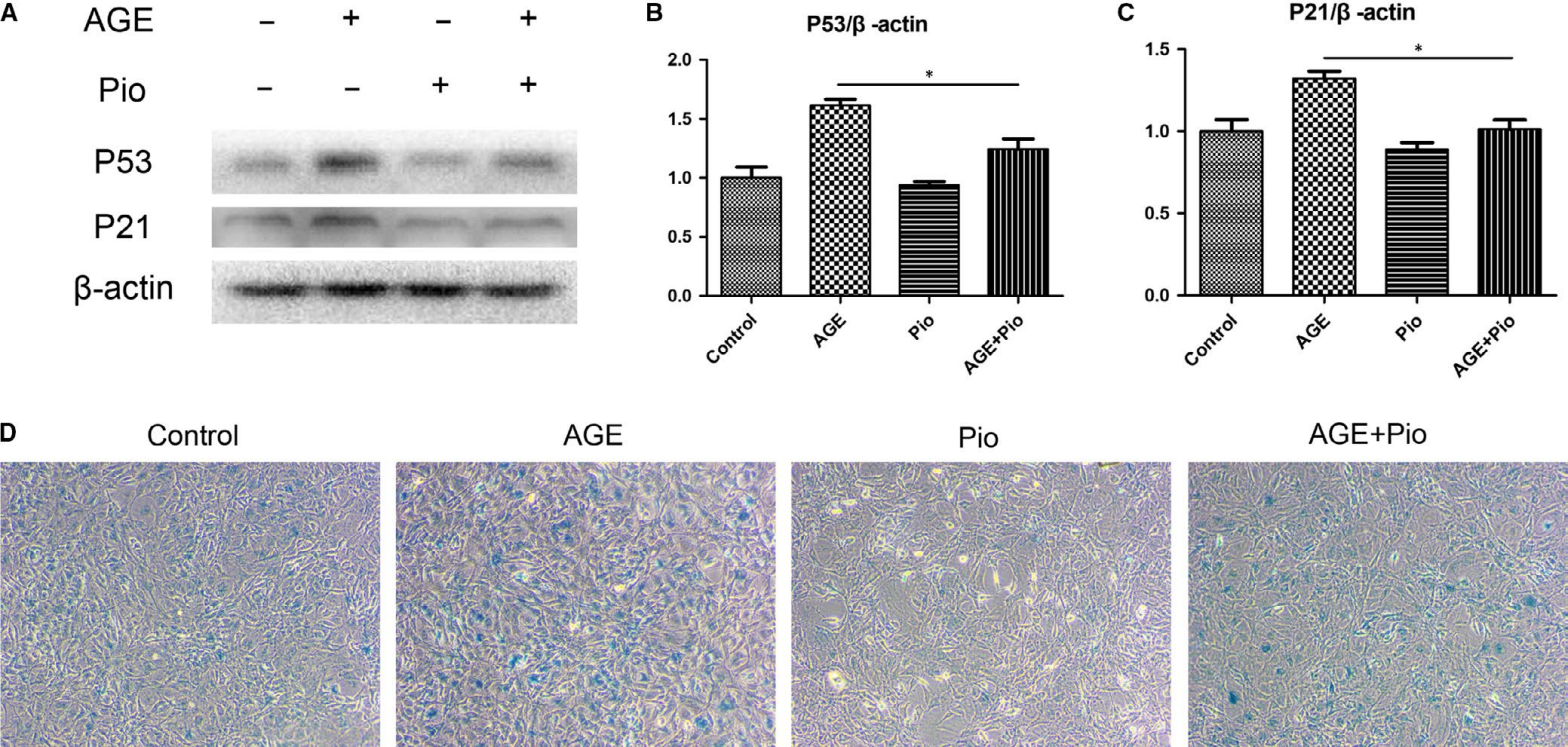
Pioglitazone alleviates AGEs‐induced senescence in TDSCs. TDSCs were pretreated with pioglitazone for 2 h and then incubated with AGEs or BSA for 24 h. (A‐C) Western blot analysis of the protein expression levels of P53 and P21 in treated TDSCs. (D) β‐galactosidase Activity Assay was performed in treated TDSCs. *P < .05

As CCK‐8 assays showed that treatment with high doses of AGEs resulted in less proliferation than treatment with control and pretreatment with Pio significantly attenuated it (Figures 2A and 5A), we next explored cellular senescence assays. Western blotting showed that expression of P53 and P21 was significantly higher in cells treated with AGEs, while Pio alleviated it (Figure 6A‐C). Treating cells with AGEs resulted in a high percentage of SA‐β‐Gal‐positive TDSCs than the cells treated with control and Pio alleviated it, too (Figure 6D). Pio therefore alleviated AGEs‐induced senescence in TDSCs.

### 3.7 Pioglitazone reverses ossification in TDSCs

**Figure 7:**
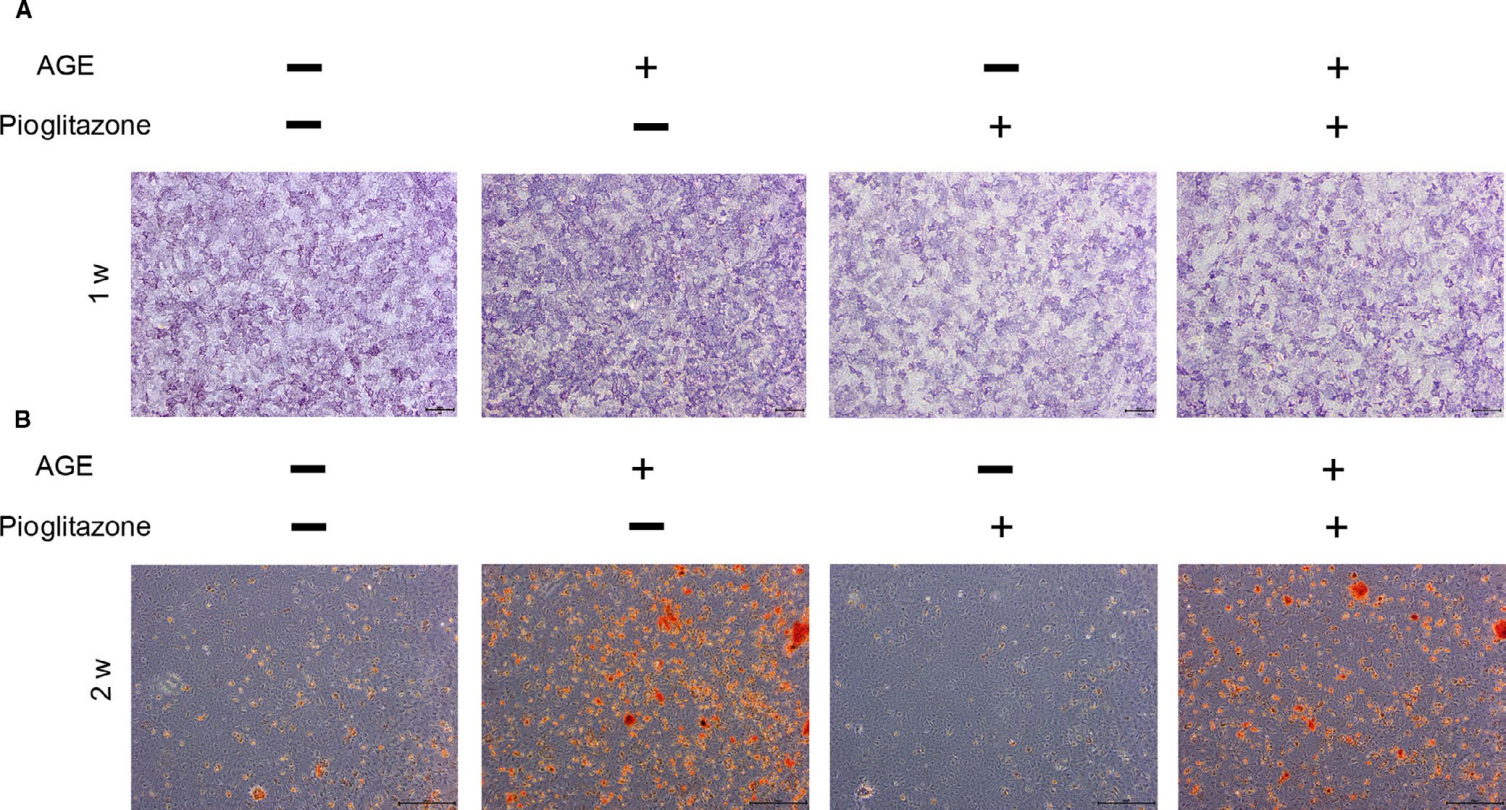
Pioglitazone reverses ossification in TDSCs. TDSCs were cultured in osteogenic induction medium along with either pioglitazone or AGEs or both. (A) ALP staining was performed in cells cultured for 5 days, Bar = 500μm. (B) Alizarin Red staining was performed in cells cultured for 14 days, Bar = 500μm

Calcific tendinopathy is a common occurrence in patients with DM who may have a mild, chronic pain condition.^21^ It is widely accepted that abnormal osteogenic differentiation of TDSCs results in the heterotopic ossification.^17^, ^22^ In this study, ALP staining and Alizarin Red staining were performed and showed that AGEs exacerbated osteogenic differentiation of TDSCs, while Pio reversed it (Figure 7A,B).

### 3.8 Pioglitazone inhibits calcification and apoptosis in vivo

**Figure 8:**
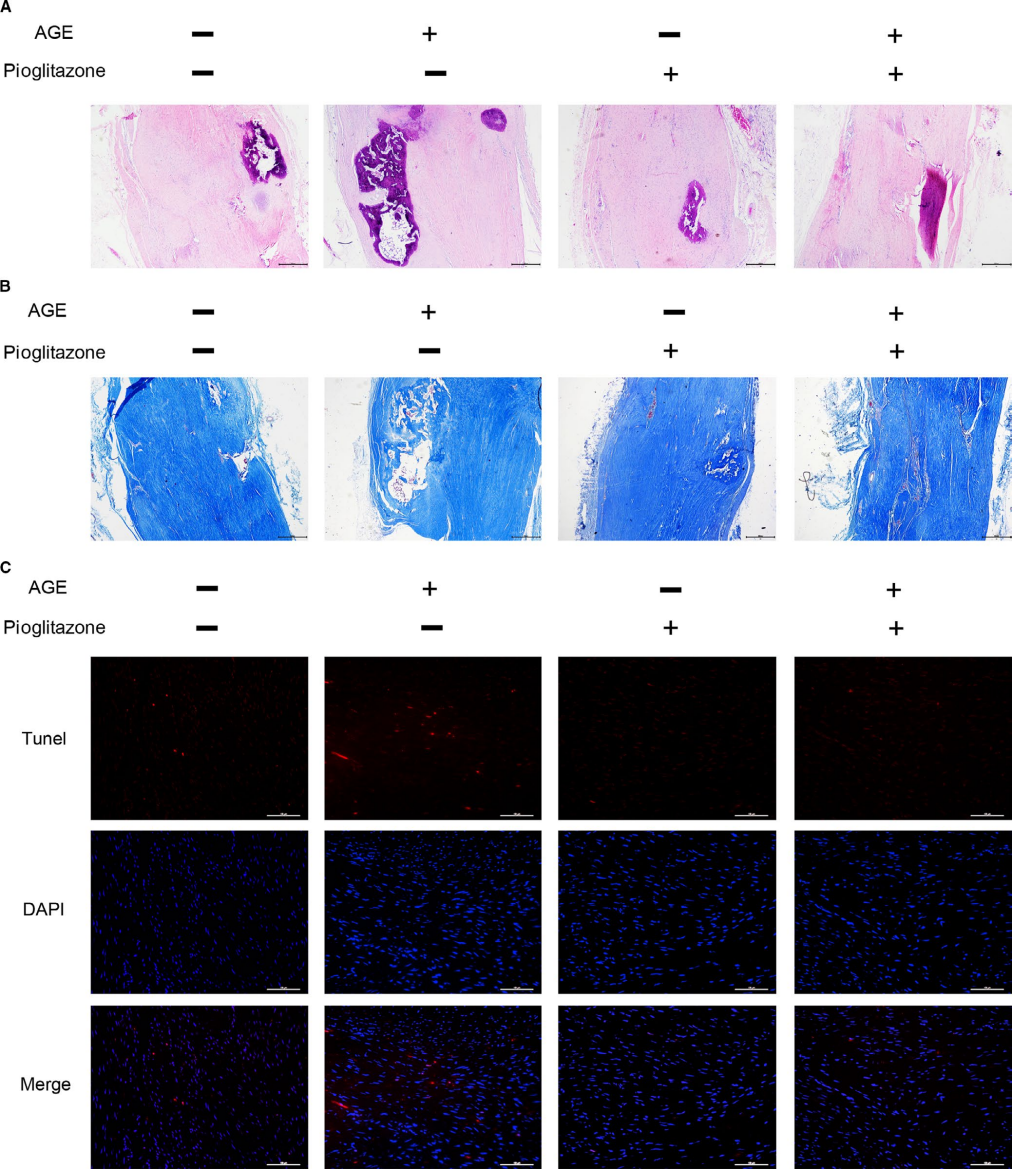
Pioglitazone inhibits calcification and apoptosis in vivo. Twenty‐four rats underwent Achilles tenotomy and administrated with AGEs/BSA and Pio/PBS through local injection for 6 weeks. (A) HE staining of Achilles's tendon from 4 groups, Bar = 500 μm. (B) Modified Masson staining of Achilles's tendon from 4 groups, Bar = 500 μm. (C) TUNEL staining of Achilles's tendon from 4 groups, Bar = 200 μm

To evaluate the effect of AGEs and Pio on rat tendon self‐healing process, HE staining and modified Masson staining were performed. We could clearly observe that the arrangement of fibroblasts and collagen fibres was obviously disordered with treatment of AGEs, while Pio reversed it. Meanwhile, AGEs administration led to more ectopic calcification in Achilles’ tendons and Pio reversed it, too (Figure 8A,B). TUNEL staining was used to evaluate the apoptosis of fibroblasts and showed that AGEs administration induced cell apoptosis, Pio protected cells against apoptosis (Figure 8C).

## 4 DISCUSSION

AGEs‐induced inflammation, oxidative stress and apoptosis are key contributors to diabetic complications, including diabetic tendinopathy. However, the underlying mechanism is still unknown. Recent research demonstrated that autophagy reversed heterotopic bone formation in tendon tissues.^17^ In this study, we demonstrated that AGEs induced TDSCs apoptosis as well as compensatory activation of autophagy. Furthermore, we confirmed that Pio‐induced activation of autophagy ameliorated the dysfunctions of TDSCs caused by AGEs.

Apoptosis, also known as programmed cell death, plays a key role in development and tissue homeostasis. However, inappropriate apoptosis is a factor in many human conditions such as neurodegenerative disease, autoimmune disorder and skeletomuscular degeneration disease.^23^ Caspases, most closely associated with apoptosis, are all capable of activating other caspases to induce a caspase cascade to execute the apoptosis process.^24^ Among all kinds of caspases, caspase‐3 is a key factor in apoptosis execution. Accumulating evidence showed that the activation of caspase‐3 was responsible for cell apoptosis in degeneration conditions.^25^ In the present study, we found that AGEs decreased cell viability, increased apoptosis rate and C‐Cas3, C‐Cas9 expression, suggesting an increase in cell apoptosis induced by AGEs.

The processes of autophagy and apoptosis modulate the balance between life and death in response to all kinds of cellular stress.^26^ Autophagy plays a key role in cell survival as well as in the regulation of cell apoptosis; numerous studies have demonstrated that promoting autophagy has protective effects against various musculoskeletal diseases such as osteoarthritis and osteoporosis.^25^, ^27^ Huang et al reported that AGEs increased the ratio of LC3B/LC3A in chondrocytes.^28^ Yang et al also reported that AGEs increased LC3B, while decreased P62 in osteoblasts.^29^ In the present study, we found a similar result in TDSCs that AGEs increased the ratio of LC3B/LC3A and decreased P62 expression, suggesting that AGEs could simultaneously induce apoptosis and autophagy. Pharmacologic activation or inhibition of autophagy was performed and showed that autophagy modulated cell apoptosis induced by AGEs, suggesting that autophagy plays a protective role in the AGEs‐induced apoptosis.

To identify whether Pio protects TDSCs, alterations in autophagy were assessed and showed that Pio increased the ratio of LC3B/LC3A and decreased P62 expression, performed as an autophagy agonist. We also confirmed that Pio promotes autophagy via modulation of the AMPK/mTOR pathway, consistent with the recent study.^13^ Further, we investigated the apoptosis of TDSCs and found that pretreatment with Pio significantly promoted cell viability, decreased apoptosis rate both in vitro and in vivo, suggesting Pio decreased AGEs‐induced apoptosis in TDSCs.

Besides, we identified that senescence of TDSCs was induced by AGEs. Senescence is defined as limiting the regenerative potential of stem cells and characterized by increased activity of SA‐β‐gal, increased G1 cell cycle arrest and expression of P53 and P21.^30^ Senescence of mesenchymal stem cells leads to a decline in their self‐renewal capacity, as well as impairing normal differentiation capacity. We found that Pio could alleviate elevated expression of P53 and P21, both of which can lead to either the exacerbate apoptosis or the induction of cell senescence.^31^ Pio could also decrease SA‐β‐Gal‐positive TDSCs, suggesting Pio alleviated AGEs‐induced senescence in TDSCs.

Interestingly, we found that AGEs enhanced ossification of TDSCs. Progression of heterotopic ossification in tendon results in restricted joint mobility and pain. The pathogenesis of heterotopic ossification is revealed to be related with osteoprogenitor stem cells in local tissue, which can be TDSCs in tendons. Abnormal ossification of TDSCs initiates osteoid formation and leads to heterotopic ossification eventually.^17^ In this study, results showed that Pio reversed exacerbated osteogenic capacity induced by AGEs both in vitro and in vivo, which provides a potential therapeutic target for heterotopic ossification prevention.

The major limitation of this study is that the mechanism by which Pio improves cellular function is not clarified thoroughly; thus, further studies on the mechanism of autophagy regulation and other related mechanisms are needed. Besides, there has been no reliable method for identifying TDSCs in vivo so far, TUNEL staining of tissue section cannot fully represent the functional status of TDSCs.

In conclusion, this study demonstrated that AGEs appear to be the primary insult and may contribute to the development of the diabetic tendon phenotype, and pioglitazone treatment induces autophagy flux in AGEs‐treated TDSCs, which possesses anti‐apoptosis, anti‐senescence and anti‐ossification effects. Thus, our study revealed the new insights to the pathophysiology of diabetic tendinitis and provided a new treatment strategy for tendinopathy.

## CONFLICT OF INTEREST

The authors declare no conflict of interest.

## AUTHOR CONTRIBUTION

All authors listed have made substantial contributions to the study. LDW, YX and LHX took part in the designing of the experiments, contributed reagents, materials and analysis tools. LHX, ZPW and KX ran the experiments. LHX, ZGC, YZH and CYM wrote the manuscript. LHX, CHZ and Safwat participated in the analysing of the data. All authors read and approved the final manuscript.

## Data Availability

All data, models and code generated or used during the study appear in the submitted article.

## References

[1] Baskerville R , McCartney DE , McCartney SM , Dawes H , Tan GD . Tendinopathy in type 2 diabetes: a condition between specialties? Br J Gen Pract. 2018;68(677):593‐594. 30498162 10.3399/bjgp18X700169 PMC6255239

[2] Lui PPY . Tendinopathy in diabetes mellitus patients‐Epidemiology, pathogenesis, and management. Scand J Med Sci Sports. 2017;27(8):776‐787. 28106286 10.1111/sms.12824

[3] Bi Y , Ehirchiou D , Kilts TM , et al. Identification of tendon stem/progenitor cells and the role of the extracellular matrix in their niche. Nat Med. 2007;13(10):1219‐1227. 17828274 10.1038/nm1630

[4] Ni M , Lui PPY , Rui YF , et al. Tendon‐derived stem cells (TDSCs) promote tendon repair in a rat patellar tendon window defect model. J Orthop Res. 2012;30(4):613‐619. 21928428 10.1002/jor.21559

[5] Shi L , Li Y‐J , Dai G‐C , et al. Impaired function of tendon‐derived stem cells in experimental diabetes mellitus rat tendons: implications for cellular mechanism of diabetic tendon disorder. Stem Cell Res Ther. 2019;10(1):27. 30646947 10.1186/s13287-018-1108-6 PMC6332703

[6] Vlassara H , Uribarri J . Advanced glycation end products (AGE) and diabetes: cause, effect, or both? Curr Diab Rep. 2014;14(1):453. 24292971 10.1007/s11892-013-0453-1 PMC3903318

[7] Luévano‐Contreras C , Gómez‐Ojeda A , Macías‐Cervantes MH , Garay‐Sevilla ME . Dietary advanced glycation end products and cardiometabolic risk. Curr Diab Rep. 2017;17(8):63. 28695383 10.1007/s11892-017-0891-2

[8] Rabbani N , Thornalley PJ . Advanced glycation end products in the pathogenesis of chronic kidney disease. Kidney Int. 2018;93(4):803‐813. 29477239 10.1016/j.kint.2017.11.034

[9] Snedeker JG , Gautieri A . The role of collagen crosslinks in ageing and diabetes ‐ the good, the bad, and the ugly. Muscles Ligaments Tendons J. 2014;4(3):303‐308. 25489547 PMC4241420

[10] Khodeer DM , Zaitone SA , Farag NE , Moustafa YM . Cardioprotective effect of pioglitazone in diabetic and non‐diabetic rats subjected to acute myocardial infarction involves suppression of AGE‐RAGE axis and inhibition of apoptosis. Can J Physiol Pharmacol. 2016;94(5):463‐476. 27119311 10.1139/cjpp-2015-0135

[11] Adeshara KA , Agrawal SB , Gaikwad SM , et al. Pioglitazone inhibits advanced glycation induced protein modifications and down‐regulates expression of RAGE and NF‐kappaB in renal cells. Int J Biol Macromol. 2018;119:1154‐1163. 30096396 10.1016/j.ijbiomac.2018.08.026

[12] Shaikh‐Kader A , Houreld NN , Rajendran NK , Abrahamse H . The link between advanced glycation end products and apoptosis in delayed wound healing. Cell Biochem Funct. 2019;37(6):432‐442. 31318458 10.1002/cbf.3424

[13] Chen W , Xi X , Zhang S , et al. Pioglitazone protects against renal ischemia‐reperfusion injury via the AMP‐activated protein kinase‐regulated autophagy pathway. Front Pharmacol. 2018;9:851. 30127742 10.3389/fphar.2018.00851 PMC6088275

[14] Hsiao PJ , Chiou HC , Jiang HJ , et al. Pioglitazone enhances cytosolic lipolysis, beta‐oxidation and autophagy to ameliorate hepatic steatosis. Sci Rep. 2017;7(1):9030. 28831172 10.1038/s41598-017-09702-3 PMC5567271

[15] Ishibashi Y , Matsui T , Takeuchi M , Yamagishi S‐I . Beneficial effects of metformin and irbesartan on advanced glycation end products (AGEs)‐RAGE‐induced proximal tubular cell injury. Pharmacol Res. 2012;65(3):297‐302. 22100460 10.1016/j.phrs.2011.11.001

[16] Huang K , Huang J , Xie X , et al. Sirt1 resists advanced glycation end products‐induced expressions of fibronectin and TGF‐beta1 by activating the Nrf2/ARE pathway in glomerular mesangial cells. Free Radic Biol Med. 2013;65:528‐540. 23891678 10.1016/j.freeradbiomed.2013.07.029

[17] Jiang H , Chen Y , Chen G , et al. Leptin accelerates the pathogenesis of heterotopic ossification in rat tendon tissues via mTORC1 signaling. J Cell Physiol. 2018;233(2):1017‐1028. 28407241 10.1002/jcp.25955

[18] Ogata Y , Mabuchi YO , Shinoda K , et al. Anterior cruciate ligament‐derived mesenchymal stromal cells have a propensity to differentiate into the ligament lineage. Regen Ther. 2018;8:20‐28. 30271862 10.1016/j.reth.2017.12.001 PMC6149186

[19] Mizushima N , Yoshimori T . How to interpret LC3 immunoblotting. Autophagy. 2007;3(6):542‐545. 17611390 10.4161/auto.4600

[20] Lee H‐M , Shin D‐M , Yuk J‐M , et al. Autophagy negatively regulates keratinocyte inflammatory responses via scaffolding protein p62/SQSTM1. J Immunol. 2011;186(2):1248‐1258. 21160040 10.4049/jimmunol.1001954

[21] Rees J , Gaida JE , Silbernagel KG , et al. Rehabilitation of tendon problems in patients with diabetes mellitus. Adv Exp Med Biol. 2016;920:199‐208. 27535262 10.1007/978-3-319-33943-6_19

[22] Shi YU , Fu Y , Tong W , et al. Uniaxial mechanical tension promoted osteogenic differentiation of rat tendon‐derived stem cells (rTDSCs) via the Wnt5a‐RhoA pathway. J Cell Biochem. 2012;113(10):3133‐3142. 22615126 10.1002/jcb.24190

[23] Elmore S . Apoptosis: a review of programmed cell death. Toxicol Pathol. 2007;35(4):495‐516. 17562483 10.1080/01926230701320337 PMC2117903

[24] Fan T‐J , Han L‐H , Cong R‐S , Liang J . Caspase family proteases and apoptosis. Acta Biochim Biophys Sin (Shanghai). 2005;37(11):719‐727. 16270150 10.1111/j.1745-7270.2005.00108.x

[25] Luo P , Gao F . The role of autophagy in chondrocyte metabolism and osteoarthritis: a comprehensive research review. Biomed Res Int. 2019;2019:1‐7. 10.1155/2019/5171602 PMC6487163 31111057

[26] Booth LA , Tavallai S , Hamed HA , Cruickshanks N , Dent P . The role of cell signalling in the crosstalk between autophagy and apoptosis. Cell Signal. 2014;26(3):549‐555. 24308968 10.1016/j.cellsig.2013.11.028 PMC4054685

[27] Wu J , Wang A , Wang X , et al. Rapamycin improves bone mass in high‐turnover osteoporosis with iron accumulation through positive effects on osteogenesis and angiogenesis. Bone. 2019;121:16‐28. 30610968 10.1016/j.bone.2018.12.019

[28] Huang W , Ao P . Autophagy protects advanced glycation end product‐induced apoptosis and expression of MMP‐3 and MMP‐13 in rat chondrocytes. Biomed Res Int. 2017;2017:1‐9. 10.1155/2017/6341919 PMC5318618 28265573

[29] Yang L , Meng H , Yang M . Autophagy protects osteoblasts from advanced glycation end products‐induced apoptosis through intracellular reactive oxygen species. J Mol Endocrinol. 2016;56(4):291‐300. 26903511 10.1530/JME-15-0267

[30] Fafian‐Labora JA , Morente‐Lopez M , Arufe MC . Effect of aging on behaviour of mesenchymal stem cells. World J Stem Cells. 2019;11(6):337‐346. 31293716 10.4252/wjsc.v11.i6.337 PMC6600848

[31] Muller M . Cellular senescence: molecular mechanisms, in vivo significance, and redox considerations. Antioxid Redox Signal. 2009;11(1):59‐98. 18976161 10.1089/ars.2008.2104

