# Supplementary figures and images for: Pioglitazone attenuates advanced glycation end products‐induced apoptosis and calcification by modulating autophagy in tendon‐derived stem cells
Source: J Cell Mol Med. 2020 Jan 19;24(3):2240–51. doi: 10.1111/jcmm.14901 (PMC7011144; doi:10.1111/jcmm.14901)

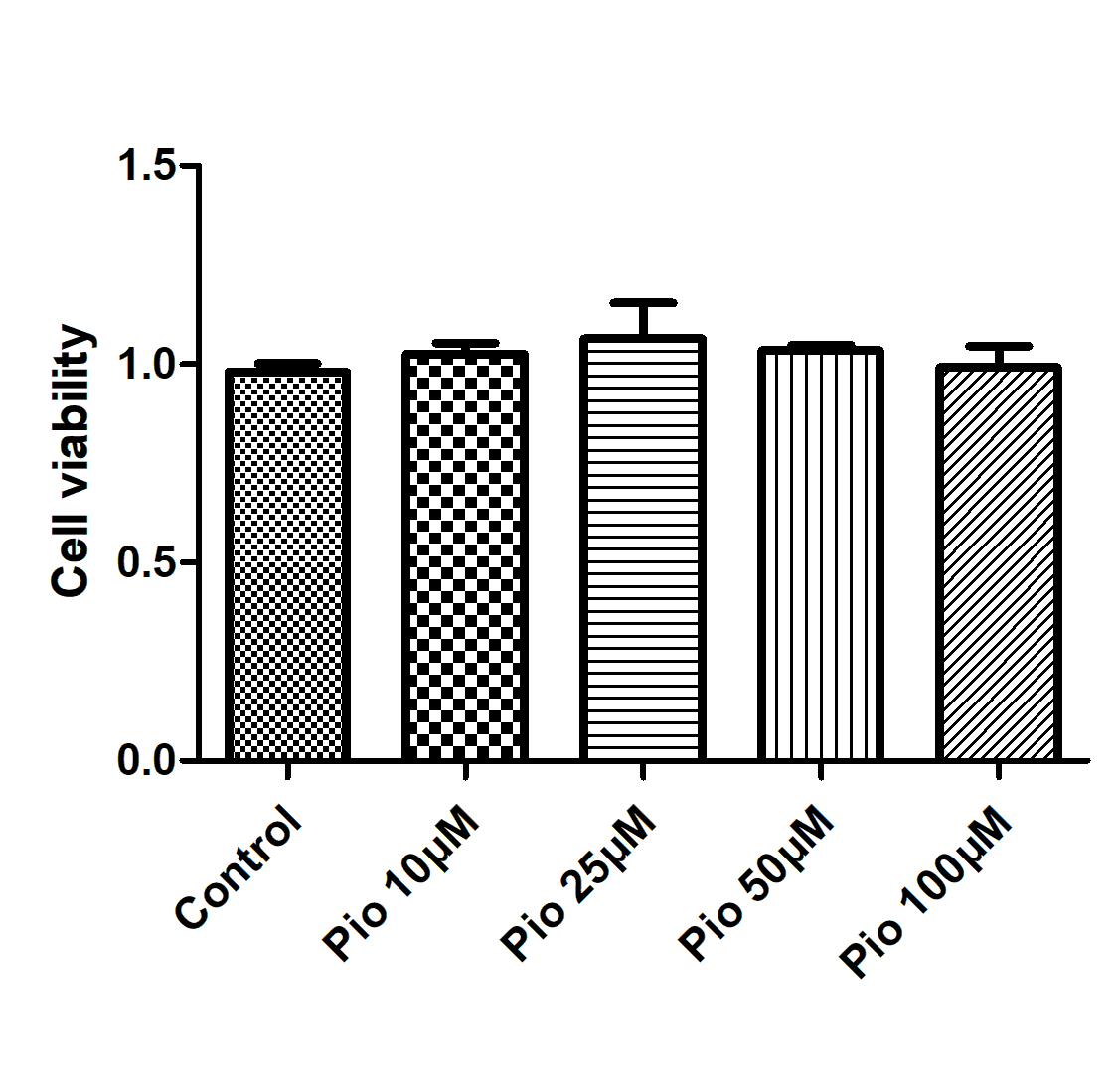

Supplement: Supplementary file 1 [file JCMM-24-2240-s001.tif]

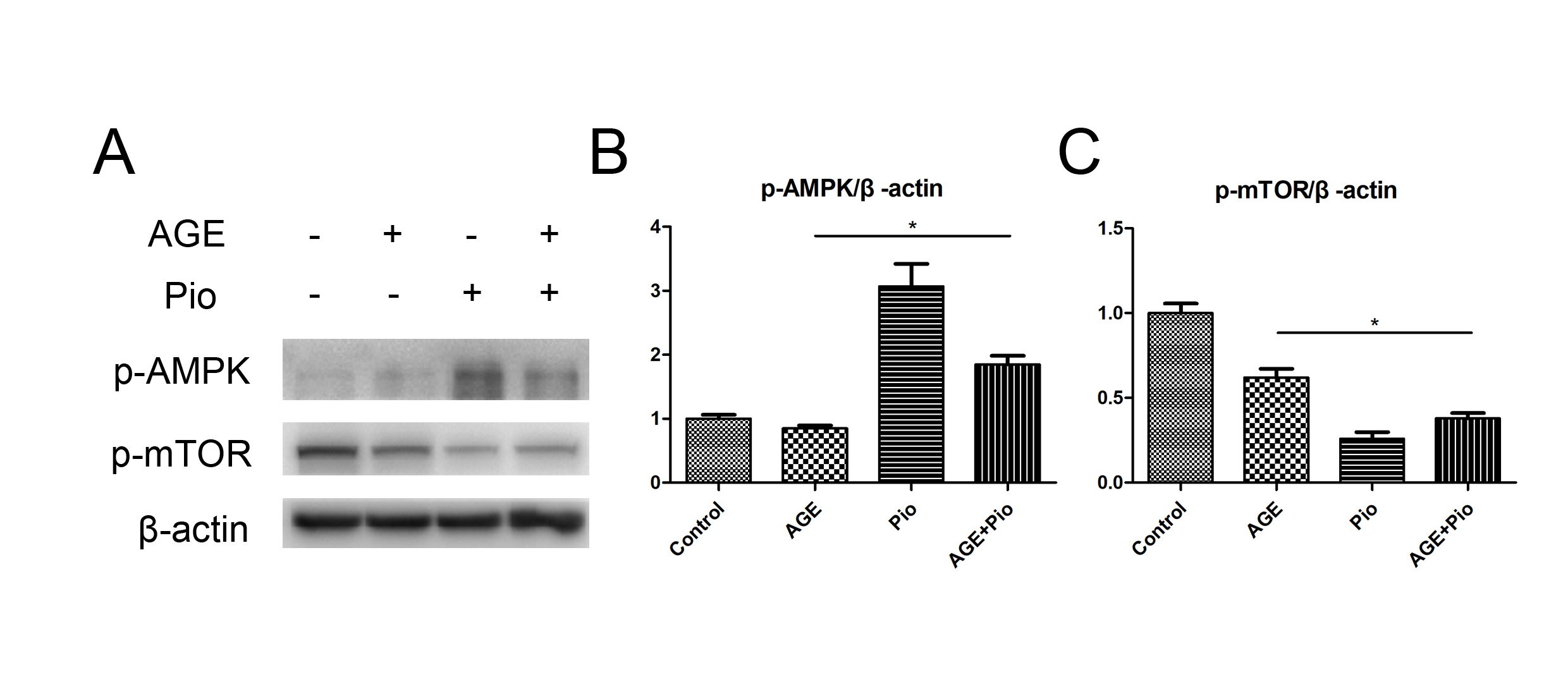

Supplement: Supplementary file 2 [file JCMM-24-2240-s002.tif]
